# Limb-salvage surgery versus extremity amputation for early-stage bone cancer in the extremities: a population-based study

**DOI:** 10.3389/fsurg.2023.1147372

**Published:** 2023-05-31

**Authors:** Yixu Zhu, Xuesong Wu, Wenjun Zhang, Haijun Zhang

**Affiliations:** Department of Orthopaedics, Xiangzhou District People’s Hospital, Xiangyang, China

**Keywords:** limb salvage, prevalence, outcomes, population based study, SEER, primary bone cancer in the extremities, extremity amputation

## Abstract

**Background:**

Many attempts have been made to induce limb salvage as an alternative to amputation for primary bone cancer in the extremities, but efforts to establish its benefits over amputation yielded inconsistent results with regard to outcomes and functional recovery. This study aimed to investigate the prevalence and therapeutic efficiency of limb-salvage tumor resection in patients with primary bone cancer in the extremities, and to compare it with extremity amputation.

**Methods:**

Patients diagnosed with T1-T2/N0/M0 primary bone cancer in the extremities between 2004 and 2019 were retrospectively identified from the Surveillance, Epidemiology, and End Results program database. Cox regression models were used to test for statistical differences between overall survival (OS) and disease-specific survival (DSS). The cumulative mortality rates (CMRs) for non-cancer comorbidities were also estimated. The evidence level in this study was Level IV.

**Results:**

A total of 2,852 patients with primary bone cancer in the extremities were included in this study, among which 707 died during the study period. Of the patients, 72.6% and 20.4% underwent limb-salvage resection and extremity amputation, respectively. In patients with T1/T2-stage bone tumors in the extremities, limb-salvage resection was associated with significantly better OS and DSS than extremity amputation (OS: adjusted HR, 0.63; 95% confidence interval [CI], 0.55–0.77; *p* < 0.001; DSS: adjusted HR, 0.70; 95% CI, 0.58–0.84; *p* < 0.001). Limb-salvage resection was associated with significantly better OS and DSS than extremity amputation for patients with limb osteosarcoma (OS: adjusted HR, 0.69; 95% CI, 0.55–0.87; *p* = 0.001; DSS: adjusted HR, 0.73; 95% CI, 0.57–0.94; *p* = 0.01). Mortality from cardiovascular diseases and external injuries was remarkably declined in primary bone cancer in the extremities patients who underwent limb-salvage resection (cardiovascular diseases, *p* = 0.005; external injuries, *p* = 0.009).

**Conclusion:**

Limb-salvage resection exhibited excellent oncological superiority for T1/2-stage primary bone tumors in the extremities. We recommend that patients with resectable primary bone tumors in the extremities undergo limb-salvage surgery as the first choice of treatment.

## 1. Introduction

Primary bone cancer is highly malignant with high mortality, particularly when the tumor metastasizing to the lung (1–3). In the United States, bone cancer has been diagnosed in approximately 3,610 new cases and resulted in 2,060 deaths in 2021(1, 4). Osteosarcoma and Ewing sarcoma have a relatively high incidence in the second decade of life, whereas chondrosarcoma is more common in older age. Osteosarcoma is the first primary cancer of bone, with an annual incidence of 0.3 per 100 000 person-years (5–8). Chondrosarcoma is the most frequent bone sarcoma of adulthood, with an annual incidence of about 0.2 per 100 000 person-years (5–8). Ewing's sarcoma is the third most common primary malignant bone tumor. Ewing's sarcoma occurs most frequently in children and adolescents, but is also seen in adults (5–8). Other types of bone cancers are less common.

The rarity of primary bone tumors has contributed to the scarcity of data on bone cancer management (9). Primary bone tumors can occur in the extremities or the trunk. Primary bone cancer in the upper extremities is attributed for 14% of all bone cancers, while primary bone cancer in the lower extremities is attributed for 37% of all bone cancers (10). The previous gold standard for surgical treatment was extremity amputation with (neo)adjuvant chemotherapy, leading to disability or emerging the need of prosthetics or allografts; however, with advances in surgical procedures, attempts have been made to introduce limb salvage as an alternative to amputation (11, 12). Since the 1980s, limb salvage has been seen as a clinically acceptable treatment for the local management of extremity bone sarcomas, which is due to improved imaging techniques and adjuvant chemotherapy. Amputation has historically been the main form of sarcoma treatment (13, 14). Amputation is still a viable option in some circumstances, such as when the tumors occur in unresectable sites, or when they are too large to be resected or spread far from their original site (15, 16).

Despite the widespread belief that radical surgery lowers the risk of recurrence and complications (17, 18), limb-salvage surgery has gained popularity among orthopedists and patients with extremity bone sarcomas due to the less physical deformity, reduced post-operation morbidities and improved functional outcomes. However, it remained unclear whether limb-salvage surgery has a negative impact on sarcoma patients’ survival (12, 19, 20). In a research comparing osteosarcoma patients treated with limb salvage with intralesional margins and those treated with limb salvage with marginal margins, 36% and 20% of patients experienced local recurrence, although no incidents were noted in patients who had their extremities amputated (21). Patients may need to have a secondary amputation, which could have a worse prognosis than main limb salvage or primary amputation, depending on the severity of the recurrence and complications after limb-salvage surgery (22, 23). However, Daniel et al. used the National Cancer Database to examine the outcomes of 2,442 patients with primary osteosarcoma in the United States, 1,855 of whom received limb-salvage surgery and 587 underwent amputation. They found that limb-salvage surgery had a significant survival advantage over amputation (24). High-quality evidence is lacking due to the rarity of bone cancer and the ethical restrictions of randomized clinical trials. Besides, the small sample sizes, distinct clinical variables, and limited scope of the available studies made it hard to draw an effective conclusion (25).

This study aimed to investigate the prevalence and outcomes of limb-salvage surgery in patients with primary bone cancer in the extremities and compare the resultant data with those of extremity amputation. In this study, we tended to depict the oncological superiority of limb-salvage resection for early-stage bone tumors in the extremities, and to demonstrate limb-salvage resection as a valuable therapeutic option for T1/2-stage bone tumors in the extremities. These results provide guidance for the management of primary bone cancer in the extremities.

## 2. Methods

### 2.1. Data sources and study population

This study extracted patients’ data from the Surveillance, Epidemiology, and End Results (SEER) program. The SEER program is a population-based framework of geographically distinct tumor registries from the US, covering approximately 30% of the US population (26). The superiority of the SEER database is that it is large-scale, real-world, and exhibits the current demographics, incidence, survival, and treatment of cancer. SEER*Stat software (version 8.3.8) was used for the analysis. All SEER data are freely accessible to researchers upon request (http://www.seer.cancer.gov) (27). This study followed the Strengthening the Reporting of Cohort Studies in Surgery reporting guidelines (28).

**Figure 1:**
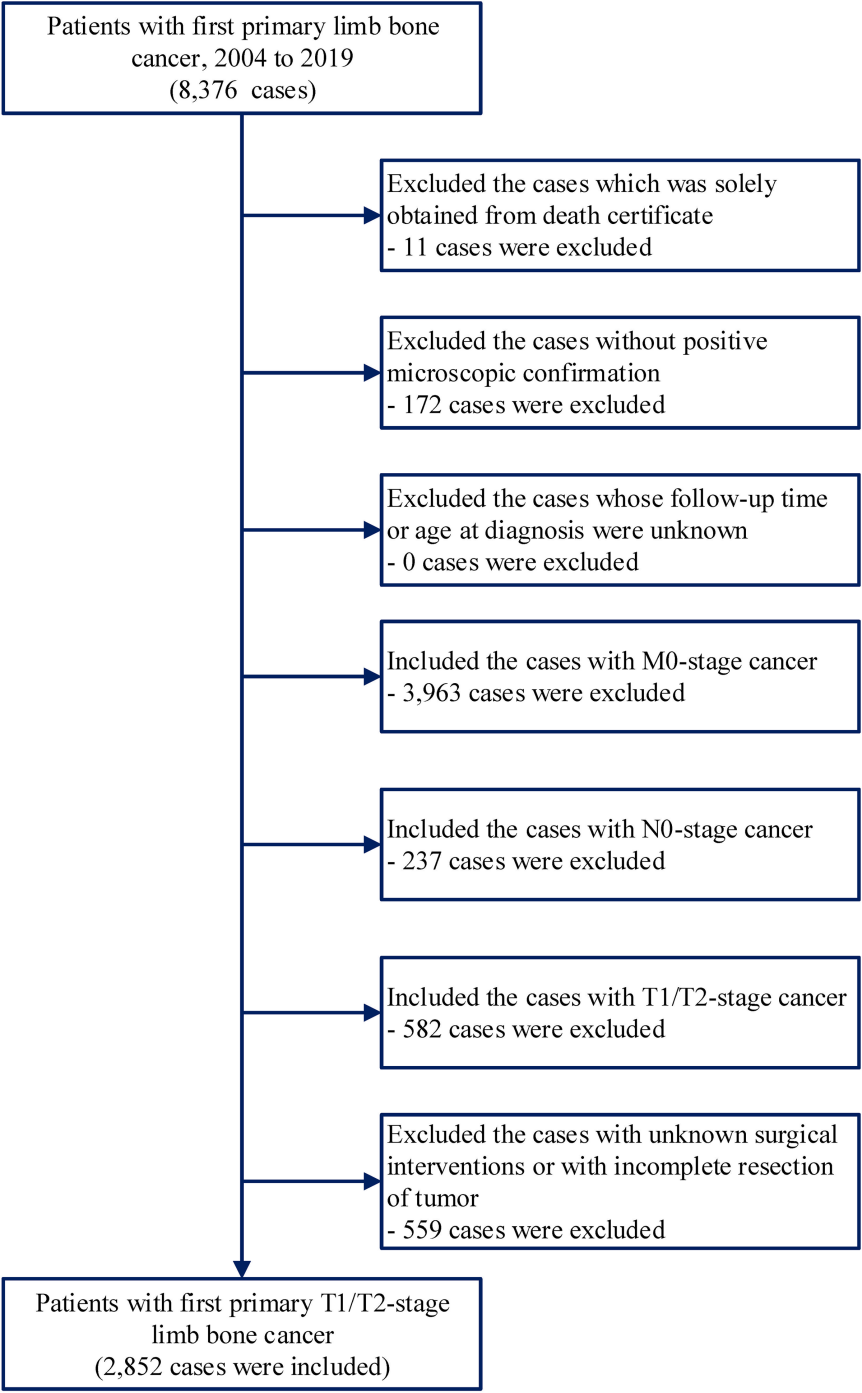
Inclusion and exclusion criteria of patients included in this study.

Data on patients with first primary bone cancer in the extremities were obtained from the National Cancer Institute's SEER program. We included first primary diagnosis of primary bone cancer in the extremities (site codes: C40.0-C40.3) during 2004–2019. We excluded patients who only had information from the death certificate. We include only early-stage bone cancer in the extremities with a stage of T1/2-N0-M0. The cases with lymph node invasion and distant metastasis were excluded. In addition, we excluded the cases with unclear surgical information or those treated by local tumor destruction (Figure 1). The data have been evaluated and checked independently by three researchers. The evidence level in this study was Level IV.

### 2.2. Definition of variables

During follow-up until December 31, 2019, relevant demographic data, tumor-specific information, type of treatment, and survival status were collected. The available patient information in the SEER database included age at diagnosis, sex, race, year of diagnosis, residential area (rural or urban), household income, and vital status at the last follow-up. Clinicopathological information for primary bone cancer in the extremities, including the AJCC TNM stage and type of surgery, was also extracted. The histology of bone cancer was defined by International Classification of Diseases for Oncology, 3rd Edition: osteosarcoma (histology codes: 9180/3–9200/3), chondrosarcoma (histology codes: 9220/3–9243/3), Ewing sarcoma (histology codes: 9260/3), and other less frequent tumors (29). Data on concomitant illnesses, such as comorbid medical and psychiatric conditions (including depression and substance abuse), were not available.

For primary bone cancer in the extremities, the T, N, and M values of the stage were based on AJCC 6th stage codes for patients diagnosed during 2004–2009, AJCC 7th stage codes for patients diagnosed during 2010–2015, and SEER combined stage for patients diagnosed during 2016–2019 (30). In the long-term survival analysis of surgery, patients were divided into three groups based on the treatment received: limb-salvage surgery, extremity-amputation surgery, and no surgical intervention (31, 32).

The causes of death in patients with primary bone cancer in the extremities were classified into two major groups: cancer- and non-cancer-related deaths (i.e., deaths from any medical cause other than cancer). The SEER cause-specific death classification variable from death certificates was used to define the causes of death (32). Non-cancer causes were categorized into seven broad categories: infectious diseases, cardiovascular diseases, respiratory diseases, gastrointestinal diseases, renal diseases, external injuries, and other non-cancer-related deaths.

### 2.3. Statistical analysis

Survival analysis was performed for overall survival (OS) and disease-specific survival (DSS) using the Kaplan–Meier method. The log-rank test was used to assess the statistical significance of survival discrepancies between the different interventions. Multivariate Cox proportional hazards models were then created to evaluate factors associated with OS and DSS. Independent variables included in the univariate model were age, race, sex, year of diagnosis, residential area, household income, AJCC T stage, and surgery type. Cumulative mortality rates (CMRs) for non-cancer comorbidities were estimated using the Kaplan–Meier method (32). For variate with multiple categories, Fisher exact test or Person's chi-squared test were adopted. A two-tailed *P *< 0.05 was considered statistically significant. Analyses were performed using the SEER*Stat software version 8.3.8 and R 3.6.3 (27, 33).

## 3. Results

### 3.1. Baseline characteristics

In this population-based study involving 2,852 patients with primary bone cancer in the extremities, 707 (24.8%) deaths were recorded, with a median follow-up time of 5.2 years (range: 0–15.9 years) (Figure 1 and Supplementary Table S1). Most of the patients were aged 0–39 years (66.5%) and were white (79.0%). Among the cancers, 46.5% and 53.5% were T1- and T2-stage tumors, respectively. Most tumors occurred in the lower extremities (78.3%), and 21.7% of them occurred in the upper extremities. 54.7% of the tumors were osteosarcoma, 26.0% were chondrosarcoma, 9.0% were Ewing sarcoma, and 10.3% were other less common tumors (Supplementary Table S1). Of the patients, 93.0% (*N* = 2,653) underwent surgical operations, among which 72.6% and 20.4% underwent limb-salvage resection and extremity amputation, respectively (Supplementary Table S1). Patients who underwent limb-salvage resection were younger (*p* < 0.001). Most patients who underwent limb-salvage resection (67.9%) were <40 years of age. Of the patients aged 0–39 years, 74.2% underwent limb-salvage resection, and only 19.1% underwent extremity amputation (Table 1). Additionally, 71.7% and 72.8% of primary bone tumors in the upper and lower extremities, respectively, were resected via limb-salvage surgery. There was a decreasing trend in the limb-salvage resection rate according to age at cancer diagnosis, especially for T1-stage tumors (Supplementary Figure S1). The black population had a higher limb-salvage resection rate (76.6%) than the white population (71.9%) (*p* = 0.009) (Table 1).

**Table 1:** Comparison on the characteristics of patients with early primary bone cancer in the extremities receiving limb salvage and extremity amputation.

Variable	Surgery		*p*
	Limb salvage	Extremity amputation	
Total	2,071 (100%)	582 (100%)	
Age			0.002
0−39	1,407 (67.9%)	362 (62.2%)	
40–59	395 (19.1%)	120 (20.6%)	
60–79	237 (11.4%)	77 (13.2%)	
80+	32 (1.5%)	23 (4%)	
Sex			0.001
Female	938 (45.3%)	218 (37.5%)	
Male	1,133 (54.7%)	364 (62.5%)	
Race			0.009
White	1,620 (78.2%)	471 (80.9%)	
Black	252 (12.2%)	55 (9.5%)	
AI/AN	17 (0.8%)	5 (0.9%)	
API	157 (7.6%)	51 (8.8%)	
Unknown	25 (1.2%)	0 (0%)	
Year			0.3
2004–2009	730 (35.2%)	226 (38.8%)	
2010–2015	839 (40.5%)	219 (37.6%)	
2016–2019	502 (24.2%)	137 (23.5%)	
Rural/urban status			0.9
Urban	1,866 (90.1%)	529 (90.9%)	
Rural	200 (9.7%)	52 (8.9%)	
Unknown	5 (0.2%)	1 (0.2%)	
Median income			0.002
Low	34 (1.6%)	2 (0.3%)	
Median	1,372 (66.2%)	420 (72.2%)	
High	665 (32.1%)	160 (27.5%)	
AJCC T stage			0.001
T1	994 (48%)	235 (40.4%)	
T2	1,077 (52%)	347 (59.6%)	
Site			< 0.001
Lower extremities	1,627 (78.6%)	474 (81.4%)	
Upper extremities	444 (21.4%)	108 (18.6%)	
Histology			0.03
Osteosarcoma	1,137 (54.9%)	350 (60.1%)	
Chondrosarcoma	565 (27.3%)	132 (22.7%)	
Ewing sarcoma	171 (8.3%)	37 (6.4%)	
Other	198 (9.6%)	63 (10.8%)	

AI/AN, American Indian/Alaska Native; API, Asian or Pacific Islander.

### 3.2. Survival analysis of surgical interventions for patients with primary bone cancer in the extremities

**Figure 2:**
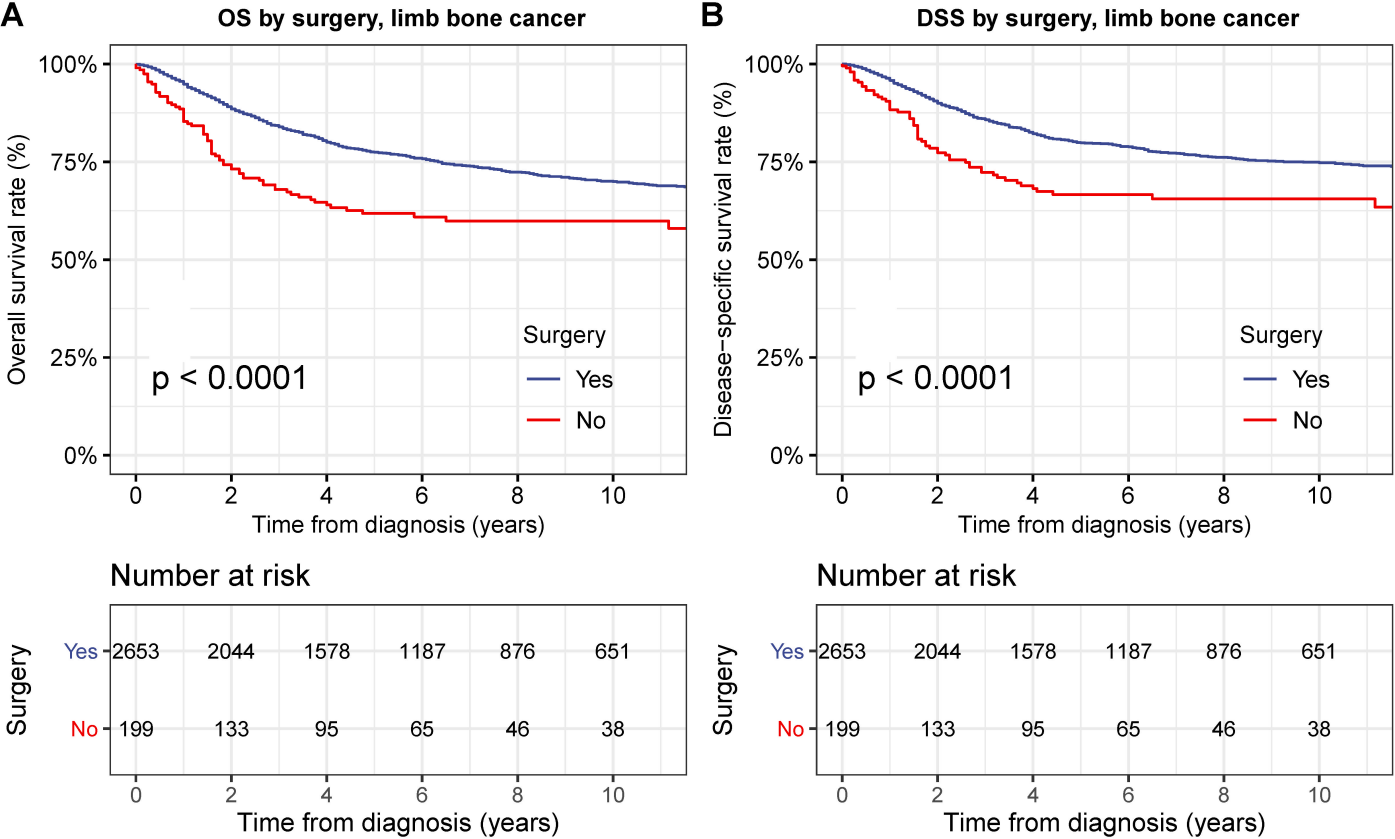
Overall survival (OS) and disease-specific survival (DSS) of patients with primary bone cancer in the extremities by surgery. (**A**) OS of patients with primary bone cancer in the extremities of all stage by surgery. (**B**) DSS of patients with primary bone cancer in the extremities of all stage by surgery.

The OS and DSS of patients who underwent surgical operations were significantly better than those who did not undergo any surgical treatment (all *p* < 0.001) (Figure 2). The 5-year DSS rate was 79.9% and 66.6% for patients who underwent and did not undergo surgical operations, respectively (Figure 2B). This prognostic superiority of OS and DSS of surgical operation could be observed in primary bone cancer in the extremities at all stages (Supplementary Figure S2).

**Figure 3:**
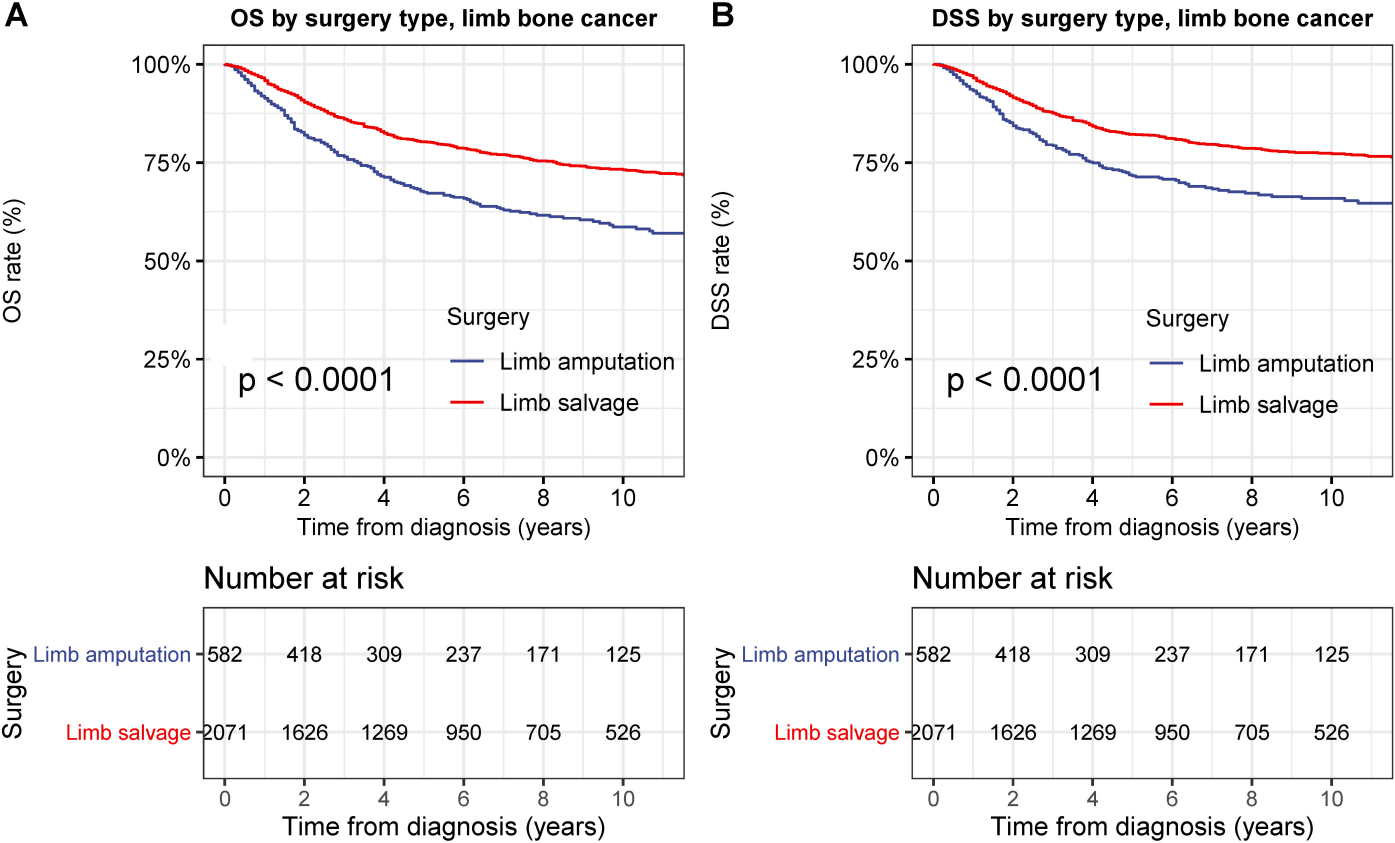
Overall survival (OS) and disease-specific survival (DSS) of patients with primary bone cancer in the extremities by different types of surgical operation. (**A**) OS of patients with primary bone cancer in the extremities of all stage by different types of surgical operation. (**B**) DSS of patients with primary bone cancer in the extremities by different types of surgical operation.

**Figure 4:**
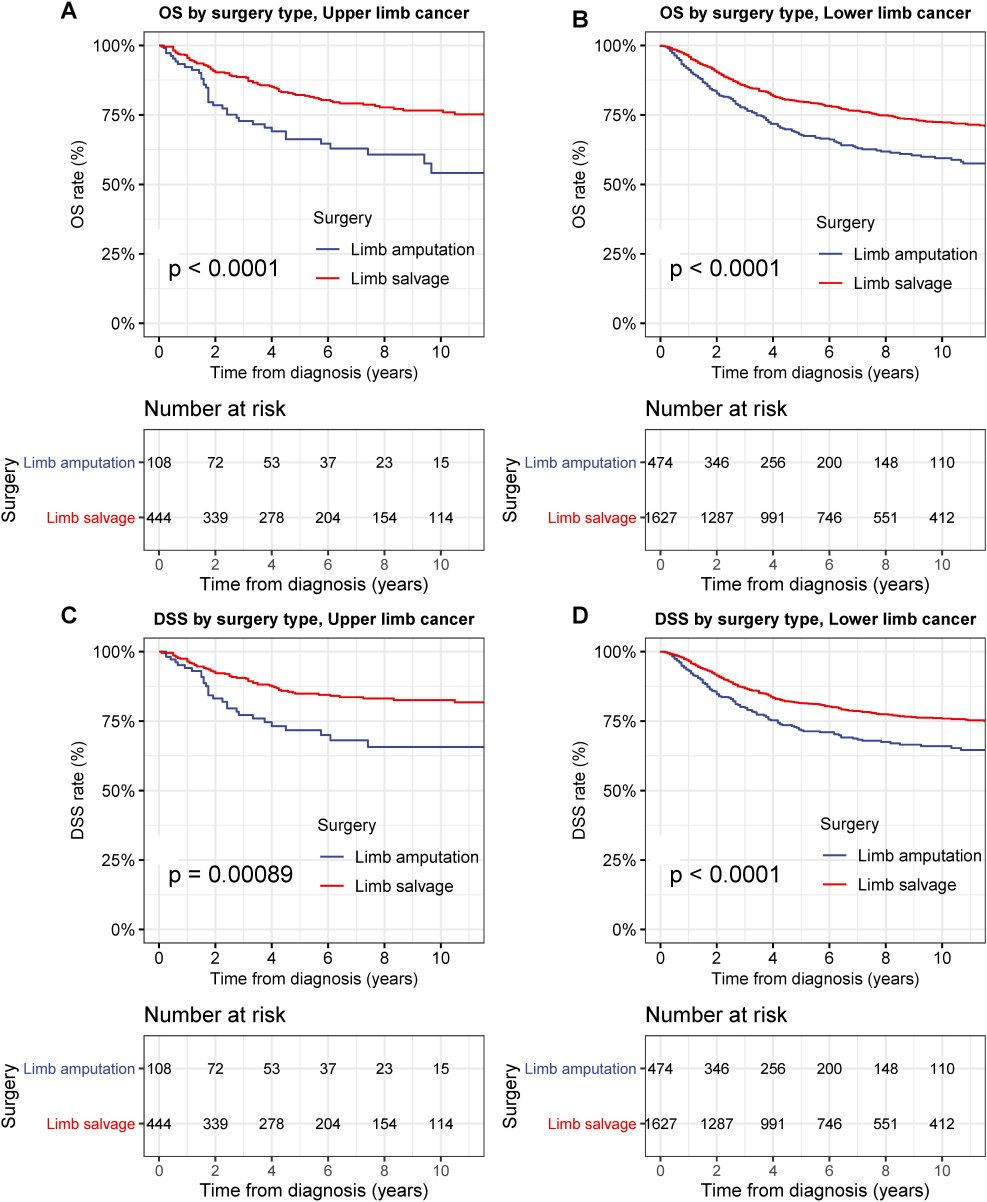
Overall survival (OS) and disease-specific survival (DSS) of patients with primary bone cancer in upper or lower extremities by different types of surgical operation. (**A**) OS of patients with primary bone cancer in the upper extremities of all stage by different types of surgical operation. (**B**) DSS of patients with primary bone cancer in the upper extremities by different types of surgical operation. (**C**) OS of patients with primary bone cancer in the lower extremities of all stage by different types of surgical operation. (**D**) DSS of patients with primary bone cancer in the lower extremities by different types of surgical operation.

To examine the therapeutic effects of limb-salvage resection, we performed survival analyses according to surgical intervention type (Figure 3). Limb-salvage resection was associated with remarkably better OS and DSS than extremity amputation (both *p* < 0.001) with a 5-year DSS rate of 82.2% for limb-salvage resection and 71.6% for extremity amputation (Figures 3A,B). The performance of limb-salvage resection was significantly better than that of extremity amputation for both upper and lower extremities (all *p* < 0.001) (Figures 4A–D).

**Figure 5:**
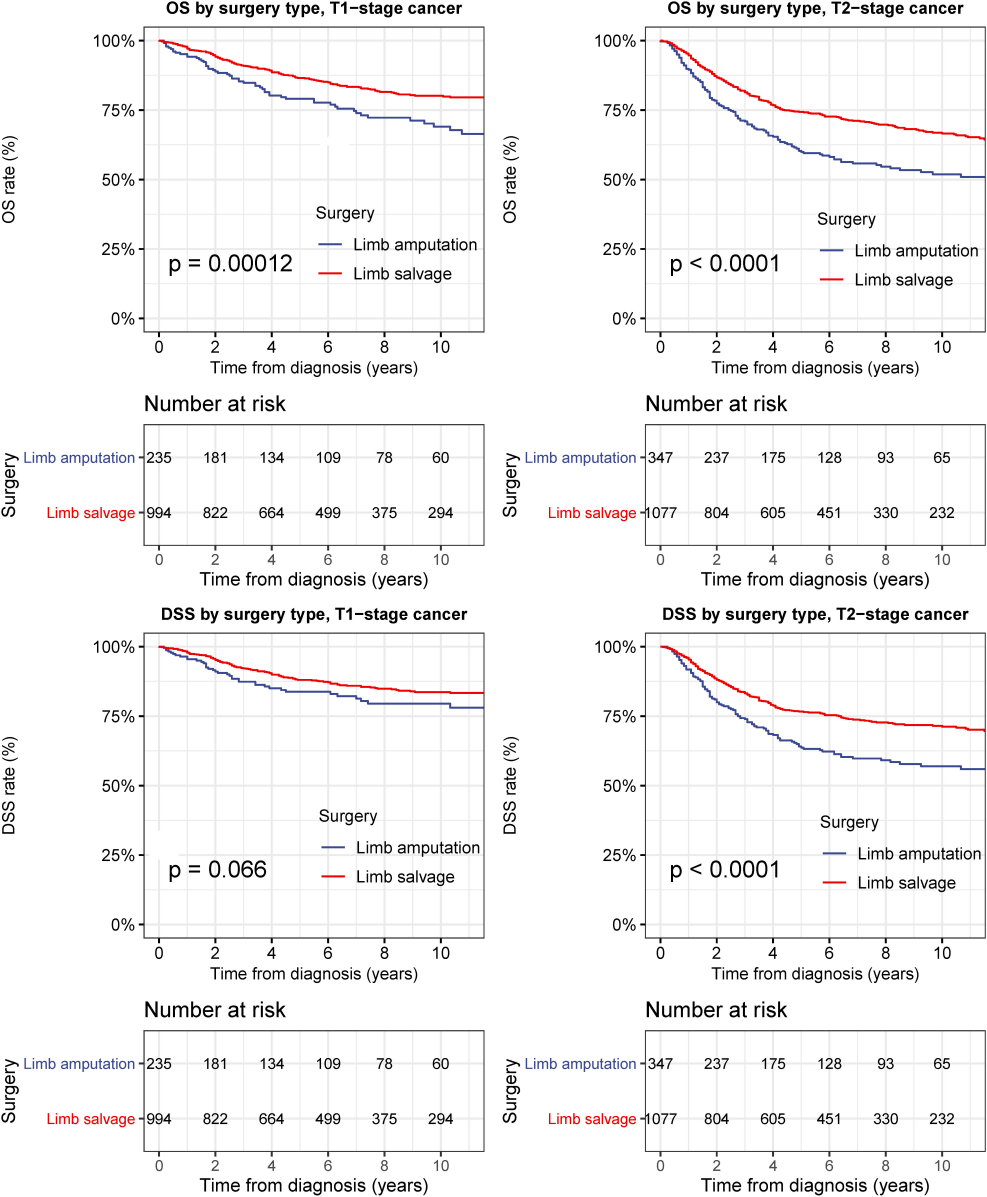
Overall survival (OS) and disease-specific survival (DSS) of patients with bone cancer in the extremities by different types of surgical operation and cancer stage. (**A**) OS of patients with T1-stage primary bone cancer in the extremities of all stage by different types of surgical operation. (**B**) DSS of patients with T1-stage bone cancer in the extremities by different types of surgical operation. (**C**) OS of patients with T2-stage bone cancer in the extremities of all stage by different types of surgical operation. (**D**) DSS of patients with T2-stage bone cancer in the extremities by different types of surgical operation.

In T1-stage bone cancer in the extremities, limb-salvage resection was also associated with remarkably better OS than extremity amputation (*p* < 0.001), with a 5-year DSS rate of 88.0% and 83.7% for limb-salvage resection and extremity amputation, respectively (Figure 5A). DSS after limb-salvage resection was slightly better than that after extremity amputation, although this did not reach statistical significance for T1-stage bone cancer in the extremities (Figure 5B). In T2-stage bone cancer in the extremities, limb-salvage resection was associated with remarkably better OS and DSS than extremity amputation (both *p* < 0.001), with a 5-year DSS rate of 76.6% for limb-salvage resection and 63.6% for extremity amputation (Figures 5C,D).

We fitted the Cox regression model to test the statistical significance of survival. To avoid the influence of patients’ baseline characteristics, tumor characteristics, and socioeconomic factors, OS and DSS analyses were performed using a Cox regression model adjusted for age at cancer diagnosis, sex, race, year of diagnosis, median household income, urban/rural residency at diagnosis, tumor site, histology type and AJCC T stage (Table 2). Limb-salvage resection was associated with significantly better OS and DSS than extremity amputation (OS: adjusted HR, 0.63; 95% confidence interval [CI], 0.55–0.77; *p* < 0.001; DSS: adjusted HR, 0.70; 95% CI, 0.58–0.84; *p* < 0.001). In multivariable Cox analyses, American Indian/Alaska Native race category (OS: adjusted HR, 2.83; *p* < 0.001; DSS, adjusted HR, 2.82; *p* < 0.001), T2-stage disease (OS: adjusted HR, 1.88; *p* < 0.001; DSS: adjusted HR, 1.99; *p* < 0.001), and older age (age group of those aged >80 years: OS: adjusted HR, 8.46; *p* < 0.001; DSS: adjusted HR, 7.17; *p* < 0.001) were predictors of grave prognosis (Table 2).

**Table 2:** Multivariate COX analyses of OS and DSS of patients with early primary bone cancer in the extremities.

Variables	OS		DSS	
	HR (95% CI)	*p*	HR (95% CI)	*P*
Surgery				
Extremity amputation	Reference		Reference	
Limb salvage	0.65 (0.55–0.77)	< 0.001	0.70 (0.58–0.84)	< 0.001
None	1.41 (1.07–1.87)	0.02	1.49 (1.1–2.03)	0.01
Age				
0–39	Reference		Reference	
40–59	1.90 (1.54–2.35)	< 0.001	1.88 (1.49–2.38)	< 0.001
60–79	4.22 (3.34–5.34)	< 0.001	4.08 (3.14–5.3)	< 0.001
80+	8.46 (5.78–12.3)	< 0.001	7.17 (4.54–11.3)	< 0.001
Sex				
Female	Reference		Reference	
Male	1.19 (1.02–1.39)	0.02	1.15 (0.98–1.37)	0.09
Race				
White	Reference		Reference	
Black	1.09 (0.86–1.38)	0.5	1.02 (0.79–1.32)	0.9
AI/AN	0.85 (0.63–1.14)	0.3	0.79 (0.57–1.09)	0.2
API	2.83 (1.4–5.73)	0.004	2.82 (1.33–5.98)	0.007
Unknown	0.36 (0.09–1.45)	0.1	0.45 (0.11–1.84)	0.3
Year				
2004–2009	Reference		Reference	
2010–2015	1.11 (0.94–1.31)	0.2	1.07 (0.9–1.28)	0.5
2016–2019	1.00 (0.77–1.31)	1	0.95 (0.71–1.27)	0.7
Median income				
Low	0.76 (0.32–1.80)	0.5	0.30 (0.07–1.23)	0.09
Median	0.86 (0.73–1.02)	0.09	0.83 (0.69–0.99)	0.04
High	Reference		Reference	
Rural/urban status				
Urban	1.11 (0.84–1.46)	0.5	1.10 (0.81–1.49)	0.5
Rural	Reference		Reference	
Unknown	0.78 (0.16–3.84)	0.8	0.43 (0.05–3.63)	0.4
AJCC T stage				
T1	Reference		Reference	
T2	1.88 (1.6–2.2)	< 0.001	1.99 (1.67–2.38)	< 0.001
Site				
Upper extremities	0.91 (0.75–1.11)	0.4	0.88 (0.71–1.1)	0.3
Lower extremities	Reference		Reference	
Histology				
Chondrosarcoma	Reference		Reference	
Ewing sarcoma	1.45 (1.02–2.05)	0.04	1.86 (1.28–2.7)	0.001
Osteosarcoma	1.89 (1.51–2.37)	< 0.001	2.24 (1.73–2.89)	< 0.001
Other	1.30 (0.99–1.7)	0.06	1.37 (1–1.88)	0.05

AI/AN, American Indian/Alaska Native; API, Asian or Pacific Islander; HR, Hazard ratios; CI, confidence interval.

**Figure 6:**
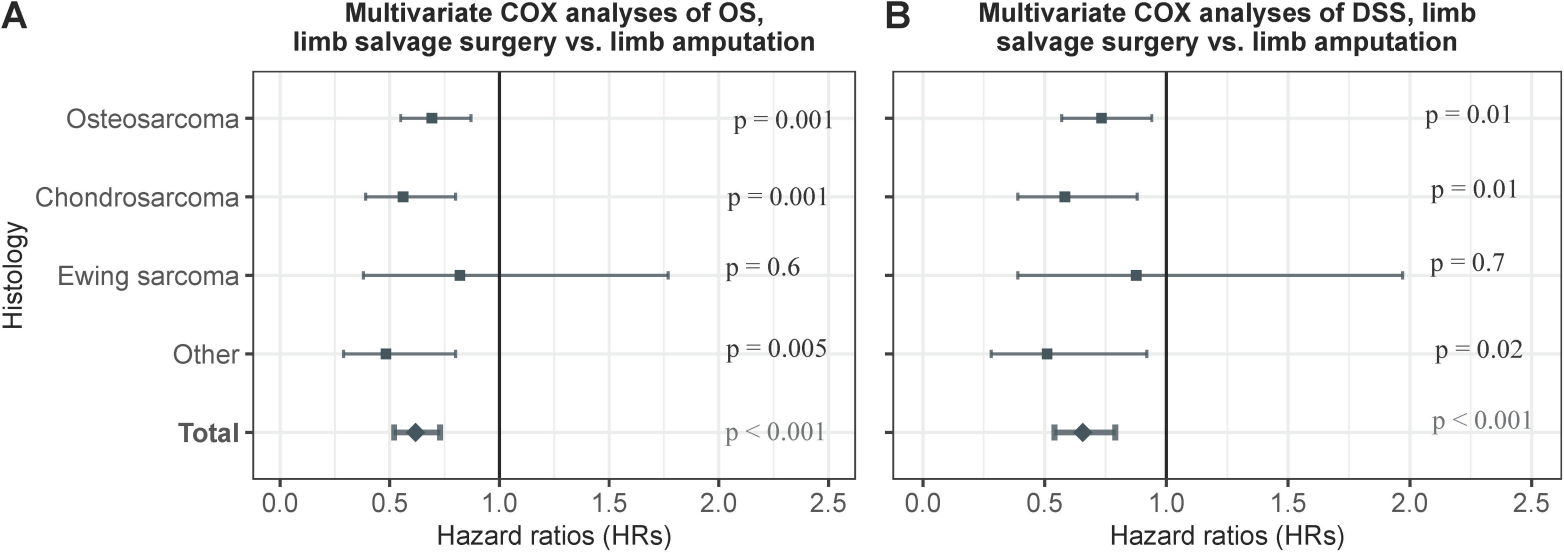
Multivariate COX analyses of OS and DSS of patients with early primary bone cancer in the extremities by histological type. (**A**) Multivariate COX analyses of OS of patients with early primary bone cancer in the extremities by histological type. (**B**) Multivariable COX analyses of DSS of patients with early primary bone cancer in the extremities by histological type.

We further analyzed the prognosis after limb-salvage resection among patients with primary bone cancer in the extremities by histology, and we found that limb-salvage resection exhibited comparable or better OS and DSS compared with extremity amputation for all types of bone cancer (Figure 6). Limb-salvage resection was associated with significantly better OS than extremity amputation for patients with osteosarcoma in the extremities (adjusted HR, 0.69; 95% CI, 0.55–0.87; *p* = 0.001), chondrosarcoma (adjusted HR, 0.56; 95% CI, 0.39–0.80; *p* = 0.001), and other bone cancer (adjusted HR, 0.48; 95% CI, 0.29–0.80; *p* = 0.004). Limb-salvage resection was associated with similar OS as extremity amputation for patients with Ewing sarcoma in the extremities (adjusted HR, 0.82; 95% CI, 0.38–1.77; *p* = 0.6) (Figure 6A). Limb-salvage resection was associated with significantly better DSS than extremity amputation for patients with osteosarcoma in the extremities (adjusted HR, 0.73; 95% CI, 0.57–0.94; *p* = 0.01), chondrosarcoma (adjusted HR, 0.58; 95% CI, 0.39–0.88; *p* = 0.01), and other bone cancer (adjusted HR, 0.51; 95% CI, 0.28–0.92; *p* = 0.02). Limb-salvage resection was associated with similar OS as extremity amputation for patients with Ewing sarcoma in the extremities (adjusted HR, 0.88; 95% CI, 0.39–1.97; *p* = 0.7) (Figure 6B).

### 3.3. Comorbidity analysis of patients with primary bone cancer in the extremities treated with different surgical interventions

**Figure 7:**
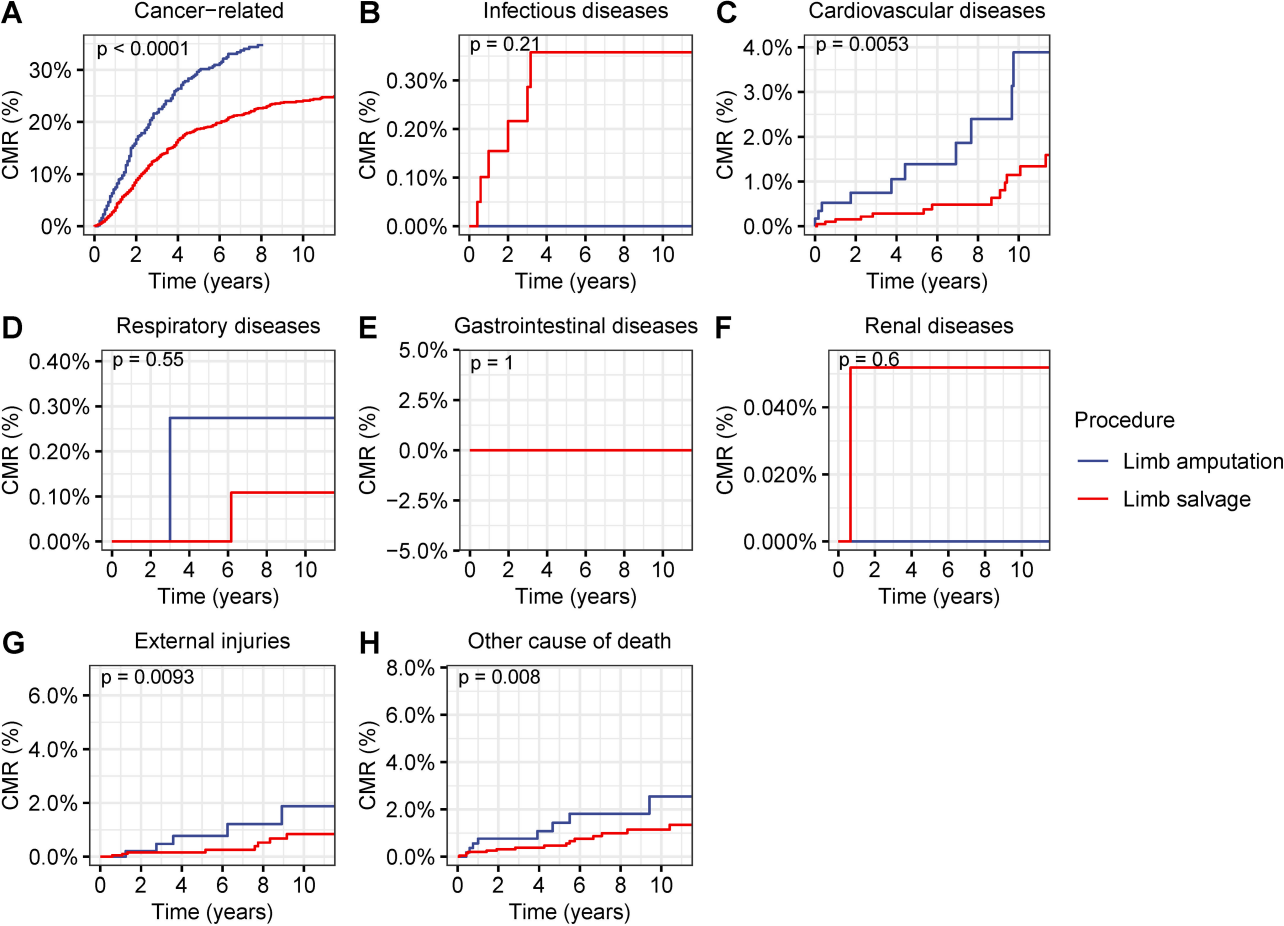
Cumulative mortality rate (CMR) among patients with primary bone cancer in the extremities by different types of surgical operation. (**A**) CMR from cancer-related deaths among patients with primary bone cancer in the extremities by different types of surgical operation. (**B**) CMR from infectious diseases among patients with primary bone cancer in the extremities by different types of surgical operation. (**C**) CMR from cardiovascular diseases among patients with primary bone cancer in the extremities by different types of surgical operation. (**D**) CMR from respiratory diseases among patients with primary bone cancer in the extremities by different types of surgical operation. (**E**) CMR from gastrointestinal diseases among patients with primary bone cancer in the extremities by different types of surgical operation. (**F**) CMR from renal diseases among patients with primary bone cancer in the extremities by different types of surgical operation. (**G**) CMR from external injuries among patients with primary bone cancer in the extremities by different types of surgical operation. (**H**) CMR from other non-cancer causes among patients with primary bone cancer in the extremities by different types of surgical operation.

Comorbidity analysis was based on the causes of death in patients with primary bone cancer in the extremities (Figure 7). In patients with primary bone cancer in the extremities, the CMR of cancer-related deaths was significantly decreased in patients receiving limb-salvage resection than in those receiving extremity amputation (*p* < 0.001) (Figure 7A). Cardiovascular deaths were remarkably high in patients who underwent extremity amputation (5-year CMR: limb-salvage resection, 0.3%; extremity amputation, 1.4%; *p* = 0.005) (Figure 7C). Mortality due to external injuries was also remarkably elevated in patients who underwent extremity amputation (5-year CMR: limb-salvage resection, 0.2%; extremity amputation, 0.8%; *p* = 0.009) (Figure 7G). Cardiovascular deaths were remarkably elevated after extremity amputation in patients with primary cancers in both upper and lower extremities (upper extremities, *p *= 0.047; lower extremities, *p *= 0.03) (Supplementary Figure S3, S4). Mortality due to external injuries was remarkably elevated after extremity amputation in patients with bone cancer in the lower extremities (*p* = 0.003) (Supplementary Figure S4G), whereas there was no significant difference in mortality from external injuries between limb-salvage surgery and extremity amputation in patients with primary cancer in upper extremities (*p* = 0.5) (Supplementary Figure S3G). When restricted the patients to younger patients aged 0–39 years, limb-salvage resection was also related with lower risks of cardiovascular deaths (*p* = 0.02) and external injuries (*p* = 0.03) (Supplementary Figure S5).

## 4. Discussion

In this study involving more than 2,800 patients with early-stage bone cancer in the extremities, we compared the prevalence and therapeutic efficiency of limb-salvage resection with that of extremity amputation. The primary purpose of this study was to compare the overall survival and disease-specific survival rates of patients with a diagnosis of primary bone cancer in the extremities undergoing limb salvage and extremity amputation surgeries for treatment. Previous studies aimed at this question have not included or evaluated primary bone cancer as one entity when evaluating outcomes in limb salvage and amputation surgery, but focus on patients with limb trauma (34–37) or all limb sarcomas (38). The results of this study exhibited an excellent oncological superiority of limb-salvage resection for early-stage bone tumors in the extremities, and suggested limb-salvage resection as a valuable therapeutic option for T1/2-stage bone tumors in the extremities.

Our results showed that limb-salvage resection was associated with a significantly better prognosis than that with extremity amputation in patients with T1- and T2-stage bone tumors in the extremities. In addition, limb-salvage resection might have the advantage of preserving the motor function and completeness of the extremities, which are completely destroyed after extremity amputation. The previous gold-standard treatment for primary bone cancer in the extremities was extremity amputation. In recent decades, great advances have been made in surgical procedures, including precise tumor location, better preoperative preparation, mastered operative skill, advanced surgical techniques, and optimal control of postoperative infections. With these advances, limb salvage tended to take the place of amputation as the dominant treatment paradigm (11, 12). In 1984, the National Institute of Health recommended limb salvage as an equal treatment option to amputation, leading to some debate regarding the future role of amputation (12, 19, 39). Fortunately, this study provided more evidence to prove that limb-salvage surgery might be a win-win strategy for the management of early primary bone cancer in the extremities.

Interestingly, we observed an elevated mortality risk for cardiovascular disease after extremity amputation in patients with early-stage bone cancer in the extremities. Previous studies have indicated that traumatic amputees may have high mortality due to CVDs (34). Post-traumatic lower extremity amputees have increased morbidity and mortality due to cardiovascular disease (35). This study revealed that tumor-induced extremity amputees might also be associated with an elevated mortality risk for CVD. Insulin resistance, psychological stress, and patients’ deviant behaviors are prevalent in extremity amputees (35). Each of these factors may have systemic consequences on the arterial system and contribute to increased cardiovascular morbidity in amputees. Individuals with dysvascular transfemoral amputation had an approximately four-fold increased risk of a cardiac event after undergoing amputation (40). Extremity amputation in patients with early-stage bone cancer in the lower extremities was associated with an increased mortality risk due to external injuries. Extremity amputation might enhance the risk of various external injuries, including falls, vehicle accidents, and self-induced injuries (suicide) (36, 37, 41–43). Lower extremity amputees are at an increased risk of falling owing to the inherent asymmetry resulting from extremity loss, muscle weakness, and other neuro-musculoskeletal limitations (41). Risk factors that increase fall in extremity amputees include lower extremity muscle weakness, increasing age, comorbidities, and the number of prescription medications (37). Studies have demonstrated a high prevalence of anxiety and depression in post-traumatic amputees, which drive some of them to commit suicide (36, 42, 43). Better body shape, self-esteem, and extremity function fetch by limb-salvage surgery will reduce the risk of postoperative anxiety, depression, falling, and other accidents.

It should be carefully evaluated before limb-salvage surgery is applied to patients with bone tumor in the extremities, particularly when the tumor is difficult to resect or when it metastasizes to distant sites. The major oncological limitations of this procedure are the underlying risks of complications from reconstructive procedures, reconstruction failure, fracture, revision surgeries, conversion to amputation after failed revision surgery, and prosthetic joint infection. Besides, there might be other oncological limitations, such as the underlying risks of residual tumor and tumor recurrence. To address these concerns, postoperative chemotherapy, radiotherapy, or molecular targeted therapy should be employed for the high-risk population. To avoid future tumor recurrence or newly developed tumors, routine screening and active follow-up should be performed after this procedure. This procedure does have its disadvantages, and amputation should be adopted when it's impossible to achieve limb salvage.

This study had several limitations. First, given its descriptive and retrospective study design, we could not prospectively assess the effects of surgical interventions in patients with primary bone cancer in the extremities and thus could not draw causal inferences (44). Second, we could not assess the physical condition, comorbidities, and other health factors of the patients. Given the high incidence of comorbidities, cognitive impairment, frailty, functional losses, social isolation, and other factors in this population, it is important to assess these factors when proposing treatment decisions; however, the SEER program did not provide this information. Third, we could not investigate the influence and the start time of specific therapies, such as radiotherapy, chemotherapy, and other targeted therapies. The SEER program only provides detailed information on surgical operations. Fourth, we cannot evaluate the functional outcome of patients who underwent limb-salvage surgery due to the lacking of functional data, which is another important prognostic indicator. Further studies with more sophisticated data should be performed to evaluate the functional outcomes after limb-salvage surgery among patients with primary bone cancer in the extremities.

Notwithstanding these limitations, this study may make an important contribution to the surgical intervention and cancer surveillance literature for cancers in the extremities. The strength of this study is that the data were derived from a high-quality population-based real-world cancer registry. The implications of this study might provide new evidence for the treatment of primary bone cancer in the extremities. Given to the limitations of this study, further prospective studies are needed to support our findings.

## 5. Conclusions

In conclusion, this study revealed that limb-salvage resection exhibited excellent oncological superiority for T1/2-stage bone tumors in the extremities. Moreover, limb-salvage resection might be associated with a reduced mortality risk from non-cancer comorbidities, including cardiovascular diseases and external injuries. Limb salvage could be a new option in recommendations of the management of physical and psychological well-being. Guidance on selecting appropriate candidates should be developed.

## Data Availability

Publicly available datasets were analyzed in this study. This data can be found here: All SEER data are freely accessible to researchers upon request (http://www.seer.cancer.gov).
