# Supplementary material for: Limb-salvage surgery versus extremity amputation for early-stage bone cancer in the extremities: a population-based study
Source: Front Surg. 2023 May 31;10:1147372. doi: 10.3389/fsurg.2023.1147372 (PMC10264616; doi:10.3389/fsurg.2023.1147372)
Supplement: Supplementary file 1 [file Datasheet1.docx]

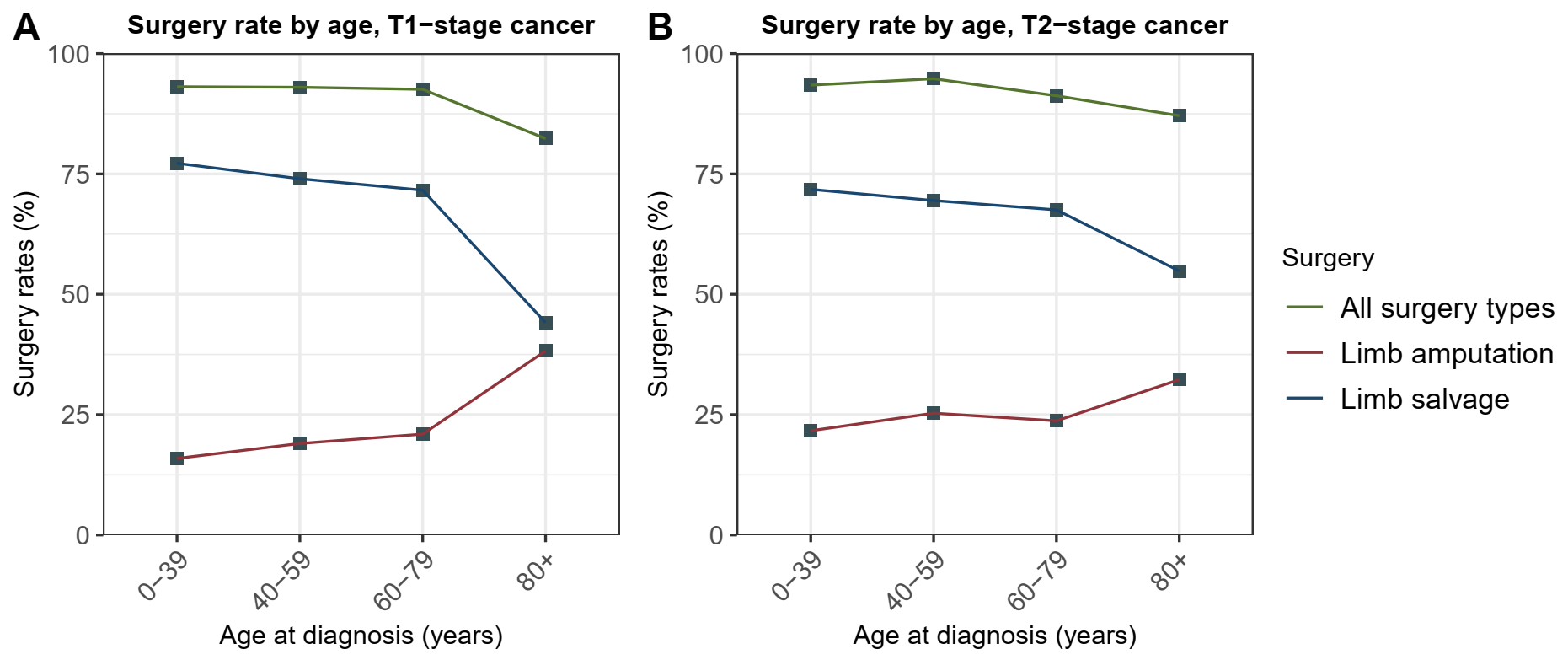


**Figure S1** Surgery rate by age at cancer diagnosis. (A) T1-stage cancer; (B) T2-stage cancer.


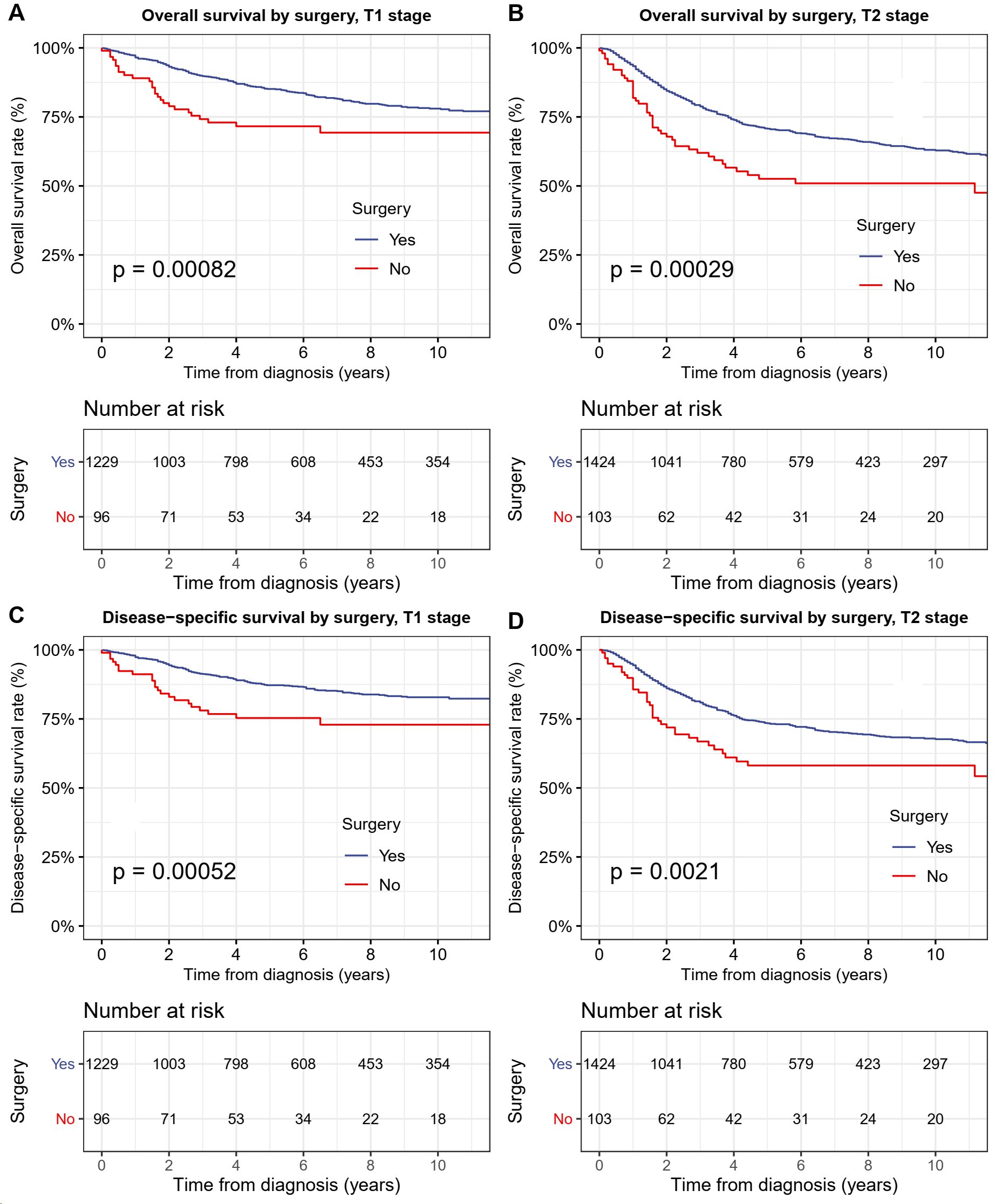


**Figure S2.** Overall survival (OS) and disease-specific survival (DSS) of patients with limb bone cancer by surgery and stage. (A) OS of patients with T1-stage limb bone cancer of all stage by surgery. (B) DSS of patients with T1-stage limb bone cancer of all stage by surgery. (C) OS of patients with T2-stage limb bone cancer of all stage by surgery. (D) DSS of patients with T2-stage limb bone cancer of all stage by surgery.


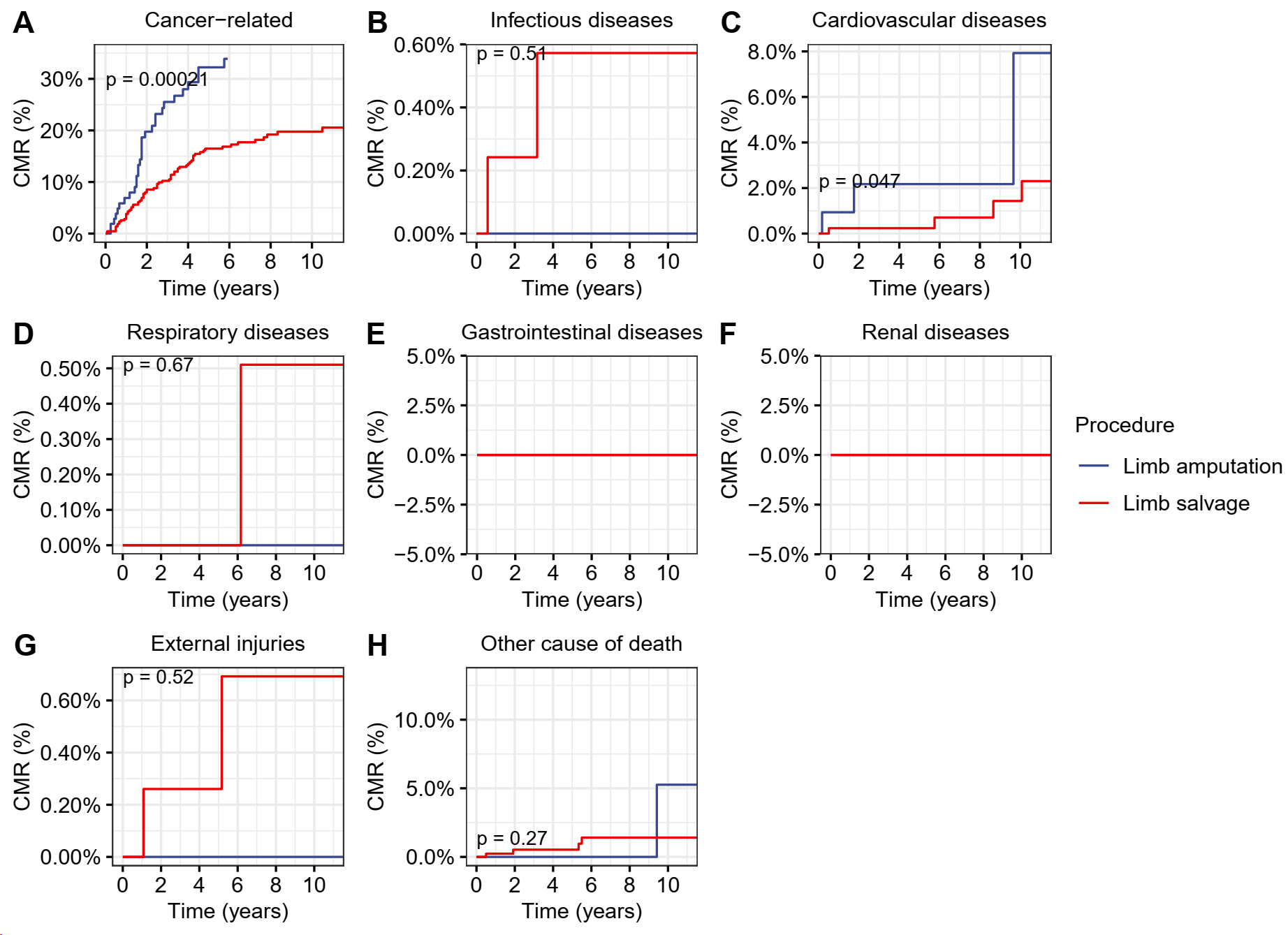


**Figure S3.** Cumulative mortality rate (CMR) among patients with upper limb bone cancer by different types of surgical operation. (A) CMR from cancer-related deaths among patients with upper limb bone cancer by different types of surgical operation. (B) CMR from infectious diseases among patients with upper limb bone cancer by different types of surgical operation. (C) CMR from cardiovascular diseases among patients with upper limb bone cancer by different types of surgical operation. (D) CMR from respiratory diseases among patients with upper limb bone cancer by different types of surgical operation. (E) CMR from gastrointestinal diseases among patients with upper limb bone cancer by different types of surgical operation. (F) CMR from renal diseases among patients with upper limb bone cancer by different types of surgical operation. (G) CMR from external injuries among patients with upper limb bone cancer by different types of surgical operation. (H) CMR from other non-cancer causes among patients with upper limb bone cancer by different types of surgical operation.


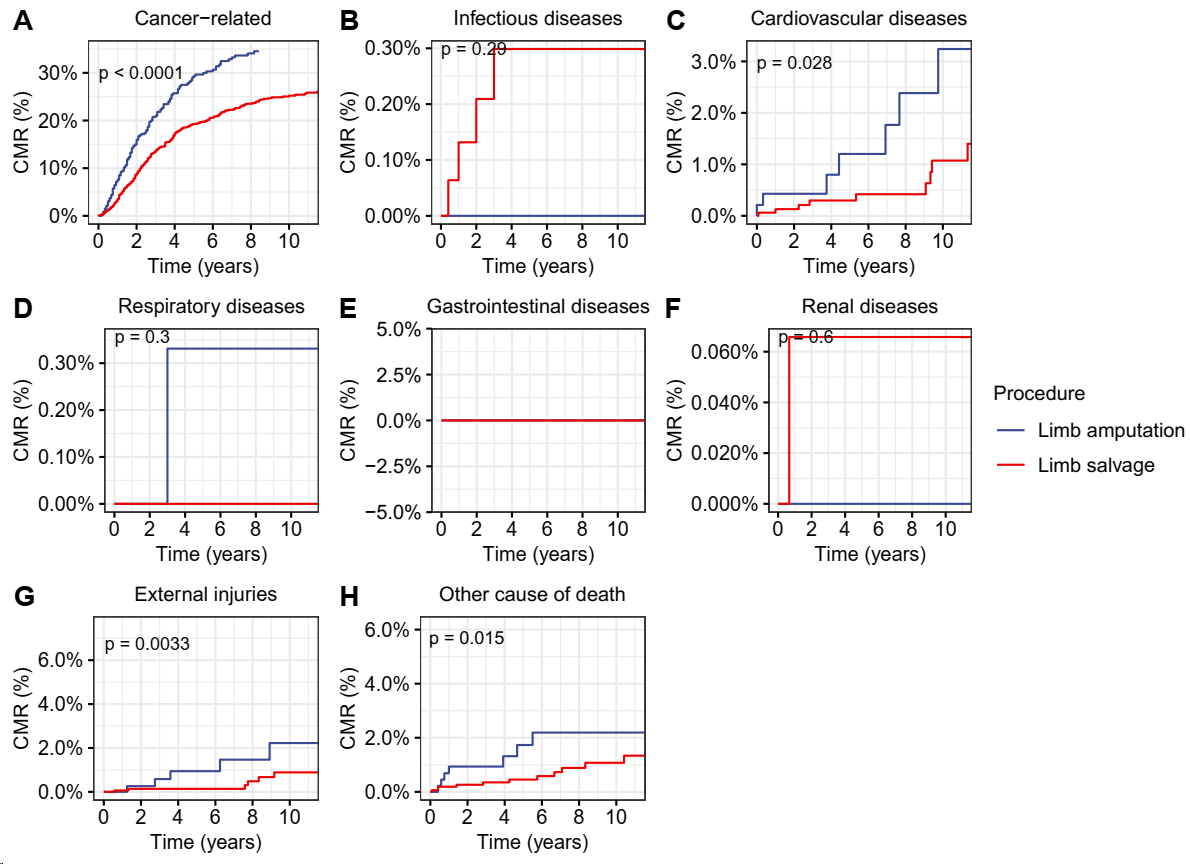


**Figure S4.** Cumulative mortality rate (CMR) among patients with lower limb bone cancer by different types of surgical operation. (A) CMR from cancer-related deaths among patients with lower limb bone cancer by different types of surgical operation. (B) CMR from infectious diseases among patients with lower limb bone cancer by different types of surgical operation. (C) CMR from cardiovascular diseases among patients with lower limb bone cancer by different types of surgical operation. (D) CMR from respiratory diseases among patients with lower limb bone cancer by different types of surgical operation. (E) CMR from gastrointestinal diseases among patients with lower limb bone cancer by different types of surgical operation. (F) CMR from renal diseases among patients with lower limb bone cancer by different types of surgical operation. (G) CMR from external injuries among patients with lower limb bone cancer by different types of surgical operation. (H) CMR from other non-cancer causes among patients with lower limb bone cancer by different types of surgical operation.


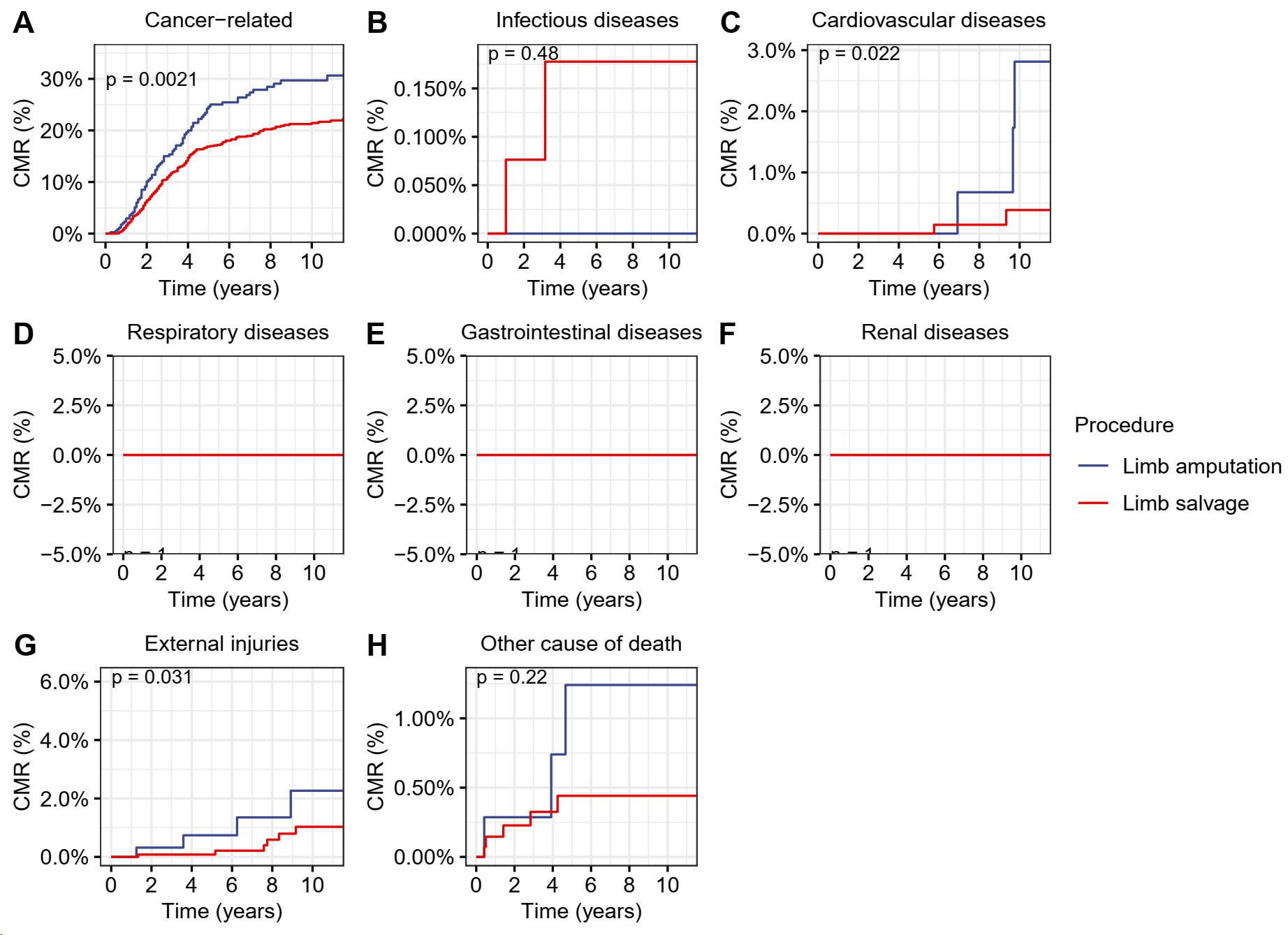


**Figure S5.** Cumulative mortality rate (CMR) among patients aged 0-39 years with limb bone cancer by different types of surgical operation. (A) CMR from cancer-related deaths among patients aged 0-39 years with limb bone cancer by different types of surgical operation. (B) CMR from infectious diseases among patients aged 0-39 years with limb bone cancer by different types of surgical operation. (C) CMR from cardiovascular diseases among patients aged 0-39 years with limb bone cancer by different types of surgical operation. (D) CMR from respiratory diseases among patients aged 0-39 years with limb bone cancer by different types of surgical operation. (E) CMR from gastrointestinal diseases among patients aged 0-39 years with limb bone cancer by different types of surgical operation. (F) CMR from renal diseases among patients aged 0-39 years with limb bone cancer by different types of surgical operation. (G) CMR from external injuries among patients aged 0-39 years with limb bone cancer by different types of surgical operation. (H) CMR from other non-cancer causes among patients aged 0-39 years with limb bone cancer by different types of surgical operation.

**Table S1** Characteristics of patients with early primary bone cancer in the extremities in the SEER program.

| Variable | No. of patients (%) | No. of deaths (%) | P^1^ |
| --- | --- | --- | --- |
| Total | 2,852 (100%) | 707 (100%) | - |
| Age |  |  | < 0.001 |
| 0-39 | 1,896 (66.5%) | 384 (54.3%) |  |
| 40-59 | 549 (19.2%) | 144 (20.4%) |  |
| 60-79 | 342 (12%) | 142 (20.1%) |  |
| 80+ | 65 (2.3%) | 37 (5.2%) |  |
| Sex |  |  | 0.02 |
| Female | 1,246 (43.7%) | 274 (38.8%) |  |
| Male | 1,606 (56.3%) | 433 (61.2%) |  |
| Race |  |  | 0.3 |
| White | 2,253 (79%) | 559 (79.1%) |  |
| Black | 329 (11.5%) | 85 (12%) |  |
| AI/AN | 23 (0.8%) | 10 (1.4%) |  |
| API | 221 (7.7%) | 51 (7.2%) |  |
| Unknown | 26 (0.9%) | 2 (0.3%) |  |
| Year |  |  | < 0.001 |
| 2004-2009 | 1,027 (36%) | 329 (46.5%) |  |
| 2010-2015 | 1,143 (40.1%) | 305 (43.1%) |  |
| 2016-2019 | 682 (23.9%) | 73 (10.3%) |  |
| Rural/urban status |  |  | 0.8 |
| Urban | 2,575 (90.3%) | 641 (90.7%) |  |
| Rural | 271 (9.5%) | 64 (9.1%) |  |
| Unknown | 6 (0.2%) | 2 (0.3%) |  |
| Median income |  |  | 0.6 |
| Low | 39 (1.4%) | 6 (0.8%) |  |
| Median | 1,933 (67.8%) | 482 (68.2%) |  |
| High | 880 (30.9%) | 219 (31%) |  |
| AJCC T stage |  |  | < 0.001 |
| T1 | 1,325 (46.5%) | 238 (33.7%) |  |
| T2 | 1,527 (53.5%) | 469 (66.3%) |  |
| Site |  |  | 0.2 |
| Lower extremities | 2,233 (78.3%) | 569 (80.5%) |  |
| Upper extremities | 619 (21.7%) | 138 (19.5%) |  |
| Histology |  |  | 0.6 |
| Osteosarcoma | 1,561 (54.7%) | 398 (56.3%) |  |
| Chondrosarcoma | 741 (26%) | 177 (25%) |  |
| Ewing sarcoma | 256 (9%) | 54 (7.6%) |  |
| Other | 294 (10.3%) | 78 (11%) |  |
| Surgery |  |  | < 0.001 |
| Extremity amputation | 582 (20.4%) | 199 (28.1%) |  |
| Limb salvage surgery | 2,071 (72.6%) | 436 (61.7%) |  |
| No surgery | 199 (7%) | 72 (10.2%) |  |

Abbreviations: AI/AN: American Indian/Alaska Native; API: Asian or Pacific Islander.
